# Pharmacogenomics of Chemically Distinct Classes of Keap1-Nrf2 Activators Identify Common and Unique Gene, Protein, and Pathway Responses In Vivo

**DOI:** 10.1124/mol.117.110262

**Published:** 2018-04

**Authors:** Ryan S. Wible, Quynh T. Tran, Samreen Fathima, Carrie H. Sutter, Thomas W. Kensler, Thomas R. Sutter

**Affiliations:** Departments of Chemistry (R.S.W., T.R.S.) and Biological Sciences (C.H.S., T.R.S.,) and the W. Harry Feinstone Center for Genomic Research (R.S.W., S.F., T.R.S.), University of Memphis, Memphis, Tennessee; Department of Preventive Medicine, University of Tennessee Health Science Center, Memphis, Tennessee (Q.T.T.); Department of Environmental Health and Engineering, Johns Hopkins Bloomberg School of Public Health, Baltimore, Maryland (T.W.K.); and Department of Pharmacology and Chemical Biology, University of Pittsburgh, Pittsburgh, Pennsylvania (T.W.K.)

## Abstract

The Kelch-like erythroid-associated protein 1 (Keap1)–NF-E2-related factor 2 (Nrf2) signaling pathway is the subject of several clinical trials evaluating the effects of Nrf2 activation on the prevention of cancer and diabetes and the treatment of chronic kidney disease and multiple sclerosis. 3*H*-1,2-dithiole-3-thione (D3T) and 1-[2-cyano-3,12-dioxooleana-1,9(11)-dien-28-oyl]imidazole (CDDO-Im) are representative members of two distinct series of Nrf2 chemical activators. Previous reports have described activator-specific effects on Nrf2-dependent gene regulation and physiologic outcomes. Here we used a robust chemical genomics approach to characterize expression profiles between D3T and CDDO-Im in livers from wild-type and Nrf2-null mice. At equally efficacious doses in wild-type mice, 406 genes show common RNA responses to both treatments. These genes enriched the Nrf2-regulated pathways of antioxidant defense and xenobiotic metabolism. In addition, 197 and 745 genes were regulated uniquely in response to either D3T or CDDO-Im, respectively. Functional analysis of the D3T-regulated set showed a significant enrichment of Nrf2-regulated enzymes involved in cholesterol biosynthesis. This result was supported by Nrf2-dependent increases in lanosterol synthase and CYP51 protein expression. CDDO-Im had no effect on cholesterol biosynthesis regardless of the dose tested. However, unlike D3T, CDDO-Im resulted in Nrf2-dependent elevation of peroxisome proliferator *α* and Kruppel-like factor 13, as well as the coactivator peroxisome proliferator *γ* coactivator 1*β*, together indicating regulation of *β*-oxidation and lipid metabolic pathways. These findings provide novel insights into the pharmacodynamic action of these two activators of Keap1-Nrf2 signaling. Although both compounds modify Keap1 to affect canonical cytoprotective gene expression, additional unique sets of Nrf2-dependent genes were regulated by each agent with enrichment of selective metabolic pathways.

## Introduction

As the key transcriptional regulator of an inducible cytoprotective response, the NF-E2-related factor 2 (Nrf2) signaling pathway is a pharmacological target for disease prevention and treatment (Deshmukh et al., 2017). The spatiotemporal regulation of Nrf2 is controlled by Kelch-like erythroid-associated protein 1 (Keap1), which facilitates the proteasomal degradation of Nrf2 (Hayes and Dinkova-Kostova, 2014). Exogenous and endogenous compounds activate Nrf2 signaling through the modification of Keap1 cysteine residues. These modifications antagonize Keap1-mediated degradation of Nrf2, thereby increasing Nrf2 protein stability and allowing for the accumulation of Nrf2 within the nucleus. Activated Nrf2 dimerizes with members of the masculoaponeurotic fibrosarcoma protein family and binds to antioxidant response elements in the promoters of target genes. Many Nrf2 target genes encode for cytoprotective enzymes such as NAD(P)H:quinone oxidoreductase 1 (NQO1) and glutathione *S*-transferase (GST), which promote cellular survival by detoxifying deleterious xenobiotic compounds and reducing oxidative stress (Kensler and Wakabayashi, 2010; Wible and Sutter, 2017).

Thus far, 10 distinct chemical classes of Nrf2-activating agents have been characterized (Kensler et al., 2007). Despite differences in their characteristic chemical structures, all 10 react favorably with cysteine sulfhydryls (Wible and Sutter, 2017). Reaction mechanisms have been best characterized for the dithiolethione and Michael acceptor classes of Nrf2 activators. Dithiolethiones, including 3*H*-1,2-dithiole-3-thione (D3T), have been shown to undergo reductive cleavage, leading to the generation of hydrogen peroxide and oxidation of sulfhydryls (Holland et al., 2009). Synthetic oleanane triterpenoids—namely, 1-[2-cyano-3,12-dioxooleana-1,9(11)-dien-28-oyl]imidazole (CDDO-Im) and congeners—covalently modify sulfhydryls through a Michael addition mechanism. Studies in zebrafish indicate that D3T specifically targets Cys151 of Keap1 (Kobayashi et al., 2009). Cys151 is also a critical sensor for CDDO-Im as determined by site-directed mutagenesis of Keap1 and the stabilization of Nrf2 protein in mice (Saito et al., 2015). Despite shared reactivity toward a single Keap1 cysteine, CDDO-Im is markedly more efficacious and potent relative to D3T. These enhanced pharmacodynamic characteristics of CDDO-Im block the formation of carcinogen-DNA adducts and prevent cancer (Kensler and Wakabayashi, 2010). Beyond Nrf2, both D3T and CDDO-Im have been shown to target transcriptional regulators and rate-limiting enzymes involved in metabolism, transcription, signal transduction, and the cell cycle (Tran et al., 2009; Liby and Sporn 2012). Along with Nrf2-dependent gene induction, the integration of these additional pathways into Nrf2 may also contribute to the desirable prophylactic and therapeutic efficacies of Nrf2-activating agents.

We and others have used chemical genomic approaches to better understand important pharmacological properties of Keap1-Nrf2 activators, including potency, chemical selectivity, structure-activity relationships within and across classes of activators, dose-response relationships, chemical versus genetic activation, and mechanisms of action (Sutter et al., 2002; Tran et al., 2009; Yates et al., 2009; To et al., 2015). Despite these advances, chemical activation of Nrf2 can result in strikingly complex compound-selective effects (e.g., disparate effects on the incidence of experimental lung cancer and distinct regulation of certain Nrf2-dependent genes) (To et al., 2015). These diverse effects of Nrf2 activation (Huang et al., 2006; Tran et al., 2009; Yates et al., 2009; To et al., 2015) likely depend on several factors, including species differences, method of pathway activation, and concentration or duration of treatment.

Considering the enormous public health potential for the prophylactic use of Nrf2-activating agents, a clear understanding of their respective pharmacodynamic properties will be critical for optimizing their therapeutic benefit. To this end, we characterized the pharmacodynamic action of two clinically relevant compounds representative of two chemical classes of Nrf2 activators, D3T and CDDO-Im. By comparing the effects elicited by each treatment administered intermittently at equally efficacious doses, we sought to control for many of the biologic and chemical variables that have confounded previous comparisons between such treatments. Combining this treatment protocol with a statistical method for clustering gene expression data into pharmacologically meaningful clusters, we identified common and unique activities of each compound at the levels of gene, protein, and pathway responses. We demonstrate that unique chemical activators of Keap1-Nrf2 modulated the common canonical Nrf2 antioxidant pathway in a manner consistent with known dose-response relationships. However, cholesterol biosynthetic enzymes were enriched by D3T, while transcriptional regulators and coactivators of lipid synthesis and *β*-oxidation were enriched by CDDO-Im. These results provide novel insights into the pharmacodynamic action of small molecule activators of Nrf2, their impact on cytoprotection, and their contributions to metabolism.

## Materials and Methods

### 

#### Chemicals.

CDDO-Im was synthesized as described previously (Honda et al., 2002). D3T was obtained from a commercial source (LKT Laboratories, St. Paul, MN).

#### Animal Care and Treatment Protocol.

All experiments were carried out in accordance with the Guide for the Care and Use of Laboratory Animals as adopted and promulgated by the U.S. National Institutes of Health and were approved by the Johns Hopkins University Animal Care and Use Committee and the University of Memphis Institutional Animal Care and Use Committee. Male mice at approximately 12 weeks of age were used for all experiments. Nrf2-null mice were produced from inbred Nrf2-heterozygous mice on a C57BL/6J background (Itoh et al., 1997; Kwak et al., 2001b). Genotypes of homozygous wild-type (Wt) and Nrf2-null mice were confirmed using polymerase chain reaction amplification of tail genomic DNA (Wakabayashi et al., 2014) (Wt primers: TGGACGGGACTATTGAAGGCTG and GCACTATCTAGCTCCTCCATTTCCGAGTC; Nrf2-null primers: GCGGATTGACCGTAATGGGATAGG and GCACTATCTAGCTCCTCCATTTCCGAGTC). Animals were housed in clear plastic cages and maintained on a 12-hour/12-hour light/dark cycle in a temperature-controlled room (24°C) with 35% relative humidity. Mice were provided an AIN-76A purified diet without ethoxyquin (Research Diets Inc., New Brunswick, NJ) ad libitum. For gene expression analyses, randomized groups of mice were treated by gavage with three doses of either 3, 10, or 30 *µ*mol/kg b.wt. CDDO-Im, 300 *µ*mol/kg b.wt. D3T, or vehicle [10% Cremophor EL (Sigma-Aldrich, St. Louis, MO), 10% dimethylsulfoxide, and 80% phosphate-buffered saline]. Treatments were administered every other day over 5 days. Mice were euthanized 24 hours after the administration of the third dose. Liver tissue was excised and freeze-clamped using liquid nitrogen. The maximal Nrf2-activating doses of each compound are known to be 30 *µ*mol/kg b.wt. CDDO-Im and 300 *µ*mol/kg b.wt. D3T (Kwak et al., 2001a; Yates et al., 2007). This dose of CDDO-Im is also the most efficacious in providing protection against carcinogenesis (Yates et al., 2006; Johnson et al., 2014).

#### RNA Isolation and Quantitative Polymerase Chain Reaction.

Total RNA was isolated from approximately 500 mg individual snap-frozen liver tissue using the RNA STAT-60 protocol (Tel-Test, Friendswood, TX) (Tran et al., 2009). RNA integrity was confirmed using an Agilent BioAnalyzer 2100 (Agilent Technologies, Santa Clara, CA). All RNA samples had an RNA integrity number greater than or equal to 8. cDNA was synthesized from 1 *μ*g total RNA using reverse transcription polymerase chain reaction. mRNA transcripts were quantified using ABsolute Blue SYBR Green quantitative polymerase chain reaction (qPCR) master mix (Thermo Fisher Scientific, Waltham, MA). Gene expression measurements were normalized to the endogenous reference gene glyceraldehyde 3-phosphate dehydrogenase, which was not affected by the treatments. Fold-change calculations were performed using the Pfaffl method (Pfaffl, 2001). Statistical analysis of qPCR results was performed as indicated in the figure legends using GraphPad Prism software (version 7.03; GraphPad Software Inc., La Jolla, CA).

#### Microarray Analysis.

Microarray data are available under Gene Expression Omnibus accession number GSE99199. An Affymetrix 3′ IVT Express Kit was used to generate and label amplified RNA (aRNA) targets (Affymetrix, Santa Clara, CA) from total RNA. aRNA was purified using the Affymetrix magnetic bead purification system. The purified product was fragmented and the labeled anti-sense aRNA was hybridized to the Affymetrix Mouse Genome 430 2.0 array. The arrays were processed as previously described. The .CEL files from the microarray chips were preprocessed by the GC-Robust Multi-array Average procedure (Wu et al., 2004). The normalized data were filtered using MAS5 calls where probe sets that had at least three present calls in any treatment group and were filtered using a 1.5-fold-change threshold. Multiple hypothesis testing was corrected using a false discovery rate (Benjamini and Hochberg, 1995) of 5%. The Kruskal–Wallis nonparametric analysis of variance, permuted 1 million times to compute exact *P* values, was used to identify differentially expressed genes by treatment. Post hoc pairwise analyses were performed using the Wilcoxon rank-sum test whereby exact *P* values were computed by a recursive procedure (Tran et al., 2009). Probe sets matching those of the significant genes identified in the Wt arrays were extracted from the Nrf2-null arrays creating genes lists containing expression values for Wt and Nrf2-null tissue as a function of treatment identity and dose. In the Affymetrix Mouse Genome 430 2.0 array chip, one gene may be represented by more than one probe set. For redundant probe sets, only those with the most numbers of smallest pairwise comparison *P* values were retained. Gene expression changes were determined to be Nrf2 dependent if one of the following criteria were met: 1) there was a statistically significant (*P* < 0.05) difference in the raw expression values between the Wt and Nrf2-null liver tissue within a treatment group or 2) there was a 30% increase or decrease in fold-change expression between the treated Wt and the treated Nrf2-null liver tissue. Genes were clustered in relation to their response to treatment (Table 1) using a hierarchical procedure described previously (Sutter et al., 2002; Tran et al., 2009). This procedure assumes a null hypothesis (H_0_) of H_0_: V = T and a two-sided alternative hypothesis of H_1_: V ≠ T, where V is the response to vehicle and T is the response to treatment, either D3T (D) or CDDO-Im (C). Genes for which H_0_ is not rejected are assigned a 1. Genes for which V > T are assigned a 0 (downregulation); where V < T (upregulation), genes are assigned a 2. A third comparison was performed to assess the relative efficacies of each treatment. The null hypothesis for this test is represented as D = C. A 1 is assigned where D = C, a 0 is assigned where D > C, and a 2 is assigned where D < C. The output of these comparisons provides a three-digit pattern (e.g., 122). The first number of this pattern indicates that the expression of this gene in response to D3T is equal to that of vehicle “1,” the response to CDDO-Im is significantly greater than that of vehicle “2,” and the response to CDDO-Im is greater than that of D3T “2.”

**TABLE 1:** The clusters of genes whose patterns identify responses to treatments

Cluster	Pattern			Genes
	VD	VC	DC	
				*n*
1	2	2	2	33
2	2	2	1	126
3	2	2	0	33
4	2	1	1	13
5	2	1	0	140
6	2	0	0	25
7	1	2	2	309
8	1	2	1	17
9	1	1	2	8
10	1	1	1	1777
11	1	1	0	18
12	1	0	1	42
13	1	0	0	377
14	0	2	2	36
15	0	1	2	36
16	0	1	1	8
17	0	0	2	9
18	0	0	1	120
19	0	0	0	85

Genes were binned into clusters based on their response to treatment. Values are assigned following the outcome of testing the null hypothesis H_0_: V = T, where V represents the vehicle and T represents the treatment. A value of 1 is assigned to comparisons in which H_0_ is not rejected. A 0 is assigned where V > T and a 2 is assigned where V < T. DC, D3T versus CDDO-Im; VC, vehicle versus 10 *μ*mol/kg b.wt. CDDO-Im; VD, vehicle versus 300 *μ*mol/kg b.wt. D3T.

#### Pathway Analysis and Enrichment.

Gene expression data from the microarray were characterized for enriched functional pathways using Ingenuity Pathway Analysis (IPA) software (Qiagen, Valencia, CA) and the Database for Annotation, Visualization and Integrated Discovery version 6.8 (DAVID) (Huang et al., 2009a,b). Kyoto Encyclopedia of Genes and Genomes (KEGG) pathway (Kanehisa et al., 2017) analysis was performed as implemented in DAVID.

#### Western Blot Protocol.

For membrane fractions, frozen liver tissue was homogenized using a Polytron homogenizer and fractionated using the Subcellular Protein Fractionation Kit for Tissues (87790; Thermo Fisher Scientific) following the manufacturer’s instructions. The expression of E-cadherin was used as a marker for the membrane fraction, as it has been shown to be localized there in mouse liver tissue (Schneider et al., 2014). Other proteins were measured in whole cell lysates from frozen liver tissue prepared by homogenizing 500 mg tissue with a Teflon pestle in 2 ml RIPA buffer [25 mM Tris, pH 7.4, 150 mM NaCl, 0.1% SDS, 0.5% sodium deoxycholate, 1% Triton X-100 and freshly added Protease Inhibitor Cocktail (Sigma-Aldrich), 1 mM phenylmethylsulfonyl fluoride, and 1 mM Na_3_VO_4_]. Lysates were cleared by centrifugation at 13,000*g* for 15 minutes at 4°C. Total protein was quantified by the Micro BCA Assay (Thermo Fisher Scientific). Fifty micrograms of either total or fractionated protein was mixed with 1× Laemmli SDS sample buffer, boiled for 5 minutes, and resolved using 10% SDS-PAGE. Proteins were blotted on polyvinylidene fluoride membranes using standard procedures. Membranes were probed using antibodies against Nqo1 (AB80588, 1:10,000; Abcam, Cambridge, MA), peroxisome proliferator *γ* coactivator 1*β* (Pgc1b) (AB176328, 1:1000; Abcam), lanosterol synthase (Lss) (13715, 1:500; Proteintech, Rosemont, IL), Cyp51 (13431, 1:500; Proteintech), sterol regulatory element-binding protein 1 (Srebp1) (sc-13551, 1:200; Santa Cruz Biotechnology, Dallas, TX), Srebp2 (sc-13552; Santa Cruz; AB30682; Abcam), protein kinase C (Pkc) (AB179521, 1:2000; Abcam), E-cadherin (610181, 1:5000; BD Biosciences, San Jose, CA), and *β*-actin (A3853, 1:12,000; Sigma-Aldrich). Secondary antibodies conjugated with horseradish peroxidase (Jackson ImmunoResearch, West Grove, PA) were used for enhanced chemiluminescent reaction (Bio-Rad, Hercules, CA). Band intensities for all blots were normalized for loading. Where indicated, band intensity was also normalized to a common sample loaded on each gel to allow for comparisons between proteins processed separately. Statistical analyses of immunoblot signals were performed as indicated in the figure legends.

## Results

**Fig. 1.**
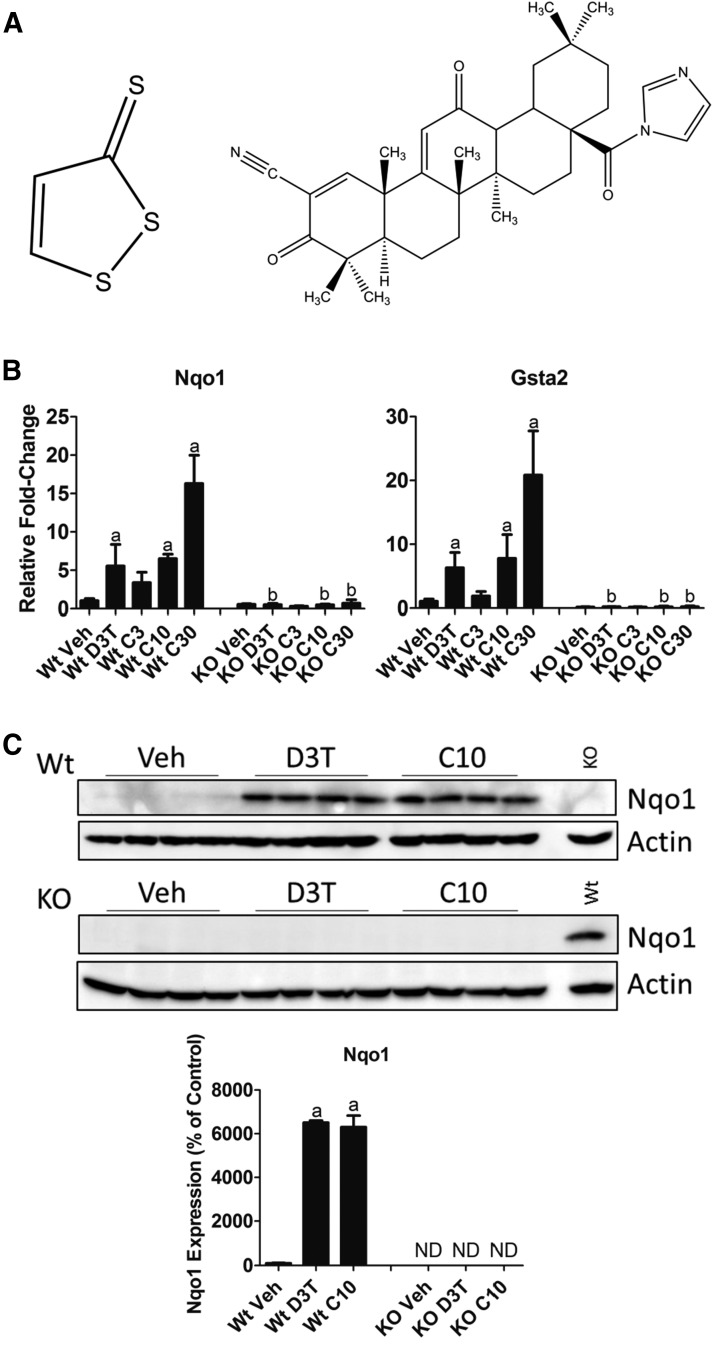
Identification of equally efficacious doses of D3T and CDDO-Im. (A) Chemical structures of D3T (left) and CDDO-Im (right). (B) Measurement by qPCR of levels of RNA of the indicated canonical Nrf2-dependent genes. Animals were treated as described in the *Materials and Methods* with doses of 300 *μ*mol/kg b.wt. D3T or 3, 10, or 30 *μ*mol/kg b.wt. CDDO-Im. Values are expressed as the average fold-change ± S.D. in the liver of Wt or Nrf2-null (KO) mice (*n* = 4/treatment group per genotype). Expression of *Gapdh*, which was unchanged by treatment or genotype, was used for normalization. Results were analyzed using a two-way ANOVA followed by Tukey’s multiple-comparisons test. ^a^*P* < 0.05 relative to the Wt vehicle (Veh); ^b^*P* < 0.05 relative to the Wt sample of the matching treatment and dose. (C) NQO1 expression in 50 *μ*g total liver protein from Wt or Nrf2-null mice treated with 300 *μ*mol/kg b.wt. D3T or 10 *μ*mol/kg b.wt. CDDO-Im. *β*-actin, which was unchanged by treatment or genotype, was used as a loading control. The lane labeled Wt in the KO blot was loaded with a 10 *μ*mol/kg b.wt. CDDO-Im–treated Wt sample as a control for antibody detection. Values are expressed as the average percentage of the Wt vehicle control, which was set to 100% ± S.D. in the liver of Wt or Nrf2-null (KO) mice (*n* = 4/treatment group per genotype). Results were analyzed using one-way ANOVA followed by Dunnett’s multiple-comparisons test. ^a^*P* < 0.05 relative to the Wt Veh. ANOVA, analysis of variance; *Gapdh*, glyceraldehyde 3-phosphate dehydrogenase; KO, knockout; ND, no protein was detected above background; Veh, vehicle.

D3T is an unsaturated five-member heterocycle with a unique carbon-sulfur double bond (Fig. 1A, left). CDDO-Im contains five 6-membered rings designated A–E (Honda et al., 1998). Functional groups of note include a carbon-carbon double bond between C1 and C2, a nitrile group on C2, a carbonyl group on C3, and an electron-withdrawing imidazolide group on C28 (Fig. 1A, right). To compare the biologic effects of D3T and CDDO-Im, we established doses of each compound that were equally efficacious in their activation of Nrf2 signaling, as determined by the induction of canonical downstream gene transcripts. Treatment of 12-week-old male mice with 300 *μ*mol/kg b.wt. D3T, the maximal tolerable dose (Kwak et al., 2001), or with 3, 10, or 30 *μ*mol/kg b.wt. CDDO-Im resulted in the upregulation of *Nqo1* and GST class *α*2 (*Gsta2*) RNA transcripts (Fig. 1B). The effects of both compounds on *Nqo1* and *Gsta2* were abrogated in Nrf2-null mice, supporting previous reports of a functional Nrf2 binding site in the promoters of *Nqo1* (Nioi et al., 2003) and *Gsta2* (Rushmore and Pickett, 1990). Based on these results, we determined 300 *μ*mol/kg b.wt. D3T to be as equally efficacious as 10 *μ*mol/kg b.wt. CDDO-Im for Nrf2 activation. To further validate this choice of equally efficacious doses, we measured NQO1 protein expression in hepatic tissue of Wt and Nrf2-null mice treated with 300 *μ*mol/kg b.wt. D3T or 10 *μ*mol/kg b.wt. CDDO-Im (Fig. 1C). Similar to the effect on RNA transcripts, the regulation of NQO1 protein by D3T and CDDO-Im was Nrf2 dependent and equal in response at these doses.

Global gene expression changes in mouse liver tissue were measured by Affymetrix microarray after treatment with equally efficacious doses of D3T and CDDO-Im. Genes were clustered using a robust nonparametric statistical procedure according to their relative responses to treatment (Table 1). Each cluster contains genes whose response(s) to treatment(s) is indicative of distinguishable pharmacological activity resulting from the presence of unique pharmacophores within the chemical structures of D3T and CDDO-Im (Sutter et al., 2002). To better assess the enrichment of functional pathways elicited by each treatment, the individual clusters of genes were collapsed into three meta clusters (Table 2). Meta cluster 1 contains genes commonly regulated by both D3T and CDDO-Im. Meta clusters 2 and 3 contain genes uniquely regulated in response to either D3T or CDDO-Im, respectively.

**TABLE 2:** Meta clusters of genes and their responses to 300 *μ*mol/kg b.wt. D3T and 10 *μ*mol/kg b.wt. CDDO-Im

Meta Cluster Number	Description	Meta Cluster Meaning	Included Gene Patterns	Genes	Nrf2-Dependent D3T	Nrf2-Dependent CDDO-Im
				*n*	*%*	
1	Common to both treatments	D3T ∼ CDDO-Im > / < Veh	22x + 00x	406	67	72
2	Unique to D3T	D3T > / < CDDO-Im ∼ Veh	21x + 01x	197	50	N/A
3	Unique to CDDO-Im	CDDO-Im > / < D3T ∼ Veh	12x + 10x	745	N/A	87

The tilde (∼)indicates that gene expression between the two treatments is not statistically different. N/A, not applicable because the pattern identifies genes that respond to only one of the treatments; Veh, vehicle.

**Fig. 2.**
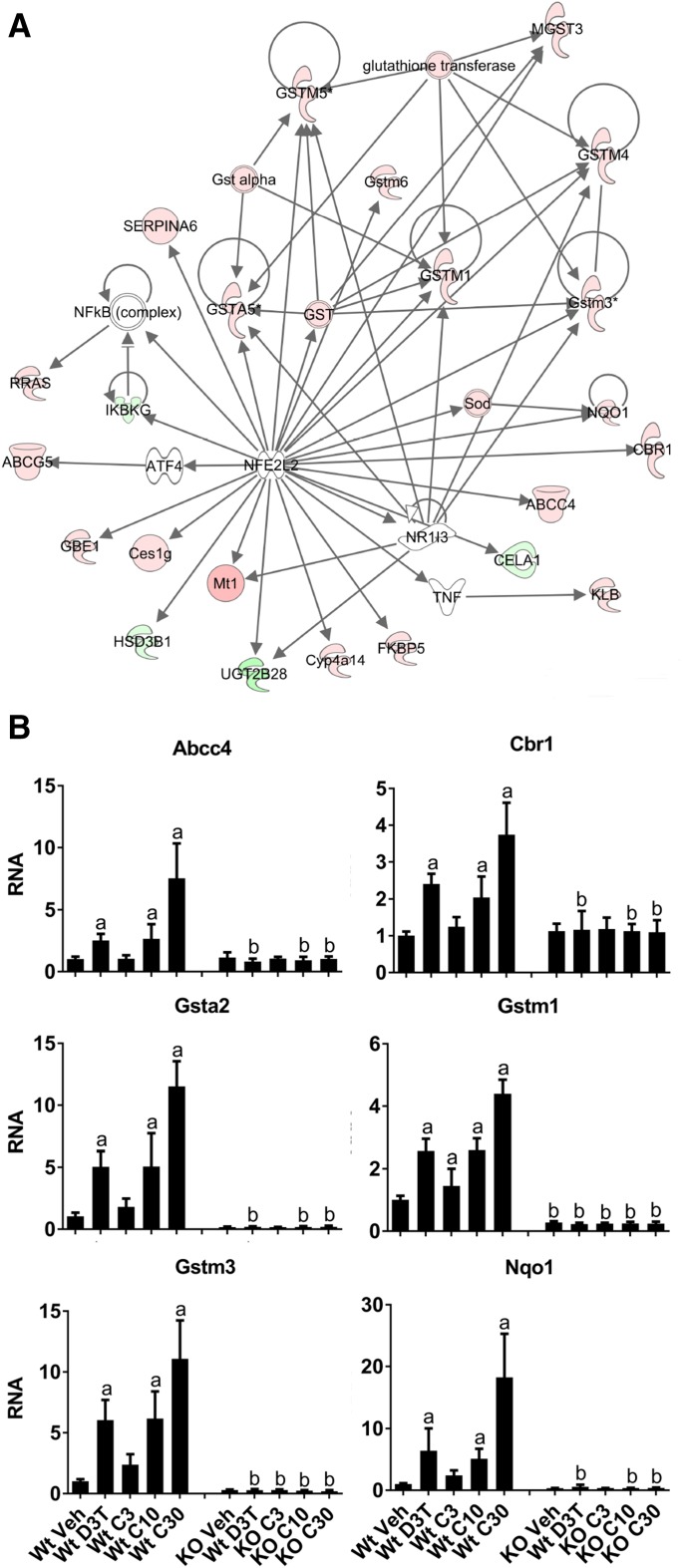
Nrf2-dependent genes with common responses to either 300 *μ*mol/kg b.wt. D3T or 10 *μ*mol/kg b.wt. CDDO-Im comprise the canonical Nrf2 antioxidant response pathway. (A) IPA for pathway enrichment of the common response genes (patterns 22x + 00x, Table 2) identified the Nrf2 antioxidant response network as the most significantly enriched. Red nodes are upregulated, and green nodes are downregulated as determined by microarray. (B) Graphical analysis of RNA levels of well studied, prototypical Nrf2-dependent genes from (A). Animals were treated as described in the *Materials and Methods* with doses of 300 *μ*mol/kg b.wt. D3T or 3, 10, or 30 *μ*mol/kg b.wt. CDDO-Im. Values are expressed as the average fold-change ± S.D. in the liver of Wt or Nrf2-null (KO) mice (*n* = 4/treatment group per genotype). Results were normalized to the expression in the Wt vehicle control, which was set to 1, and analyzed using a two-way analysis of variance followed by Tukey’s multiple-comparisons test. ^a^*P* < 0.05 relative to the Wt vehicle; ^b^*P* < 0.05 relative to the Wt sample of the matching treatment and dose. KO, knockout; Veh, vehicle.

Meta cluster 1 (Table 2) contains 406 genes that are commonly regulated by both D3T and CDDO-Im, with the majority of them determined to be dependent on Nrf2 for regulation (Wt vs. Nrf2-null comparisons). IPA functional analysis of these genes identified the canonical Nrf2 antioxidant response network as highly enriched (Fig. 2A). Graphical analysis of several well characterized, prototypical Nrf2-dependent genes identified by microarray, including ATP-binding cassette, subfamily C member 4 (Yates et al., 2009), carbonyl reductase 1 (Agyeman et al., 2012), *Gsta2*, *Gstm1* and *Gstm3* (Chanas et al., 2002; Malhotra et al., 2010), and *Nqo1* (Chorley et al., 2012), provides further confirmation that 300 *μ*mol/kg b.wt. D3T and 10 *μ*mol/kg b.wt. CDDO-Im are equally efficacious activators of canonical Nrf2 signaling (Fig. 2B). Moreover, these data also demonstrate that a higher dose of CDDO-Im (30 *μ*mol/kg b.wt.) is a more effective inducer of Nrf2 compared with the maximal dose of D3T (approximately 2-fold higher responses).

Meta cluster 2 contains 197 genes that respond only to D3T (Table 2). Only 98 genes (50%) in this cluster were dependent on Nrf2. In support of our classification of these genes as unique to D3T, we evaluated the response characteristics of the 72 Nrf2-dependent genes that were upregulated in response to D3T in this cluster. Only 18 of these genes (25%) showed any level of response to CDDO-Im above 1.25-fold, a cutoff value that our laboratory can routinely verify by qPCR methods (Kennedy et al., 2013). In contrast, 54 genes (75%) were only regulated by D3T. These data indicate that the genes in meta cluster 2 are truly unaffected by CDDO-Im and did not just fail to reach statistical significance.

**Fig. 3.**
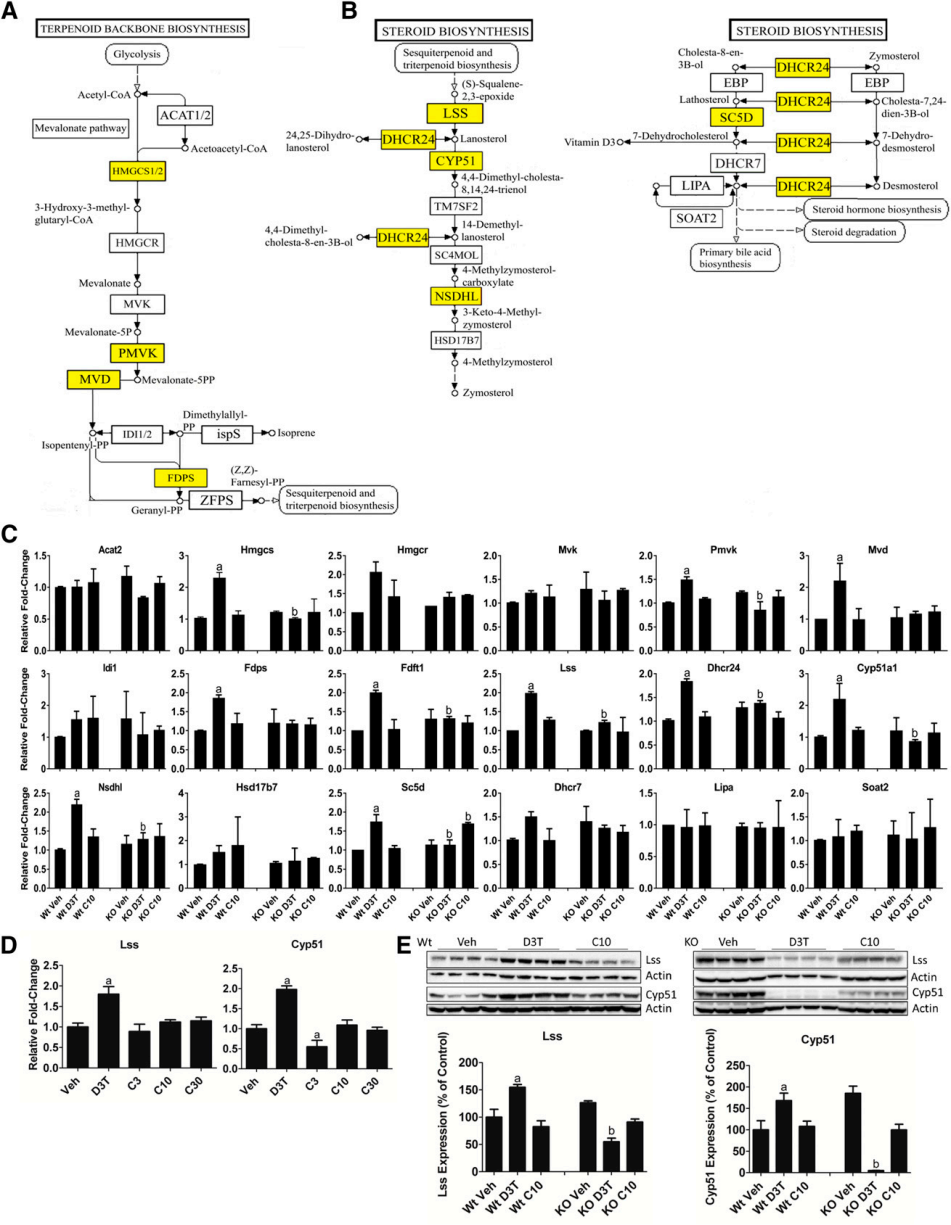
D3T uniquely upregulates the cholesterol biosynthesis pathway in mouse liver. (A and B) KEGG pathways of terpenoid backbone synthesis (A) and cholesterol biosynthesis (B) are a significantly enriched set of genes uniquely responsive to D3T (patterns 21x + 01x, Table 2). Yellow highlighted squares identify genes that are upregulated uniquely by D3T as determined by qPCR in (C). (C) Measurement by qPCR of levels of RNA of the indicated cholesterol biosynthetic enzymes. Animals were treated as described in the *Materials and Methods* with doses of 300 *μ*mol/kg b.wt. D3T or 10 *μ*mol/kg b.wt. CDDO-Im. Values are expressed as the average fold-change ± S.E.M. in the liver of Wt or Nrf2-null (KO) mice (*n* = 2 independent experiments of four animals/treatment group per genotype each). Expression of *Gapdh*, which was unchanged by treatment or genotype, was used for normalization. Results were analyzed using a two-way ANOVA followed by Tukey’s multiple-comparisons test. ^a^*P* < 0.05 relative to the Wt vehicle; ^b^*P* < 0.05 relative to the Wt sample of the matching treatment and dose. (D) Measurement by qPCR of levels of RNA of *Lss* and *Cyp51*. Animals were treated as described in the *Materials and Methods* with doses of 300 *μ*mol/kg b.wt. D3T or 3, 10, or 30 *μ*mol/kg b.wt. CDDO-Im. Values are expressed as the average fold-change ± S.D. in the liver of Wt mice (*n* = 4/treatment group per genotype). Expression of *Gapdh*, which was unchanged by treatment or genotype, was used for normalization. Results were analyzed using one-way ANOVA followed by Dunnett’s multiple-comparisons test. ^a^*P* < 0.05 relative to the Wt vehicle. (E) LSS and CYP51 expression in 50 *μ*g total liver protein from Wt or Nrf2-null mice treated with 300 *μ*mol/kg b.wt. D3T or 10 *μ*mol/kg b.wt. CDDO-Im. *β*-actin, which was unchanged by treatment or genotype, was used as a loading control and then data were normalized to a common sample loaded on both gels to allow for comparisons between genotypes. Values are expressed as the average percentage of the Wt vehicle control, which was set to 100% ± S.D. in the liver of Wt or Nrf2-null (KO) mice (*n* = 4/treatment group per genotype). Results were analyzed using a two-way ANOVA followed by Tukey’s multiple-comparisons test. ^a^*P* < 0.05 relative to the Wt vehicle; ^b^*P* < 0.05 relative to the Wt sample of the matching treatment and dose. ANOVA, analysis of variance; *Gapdh*, glyceraldehyde 3-phosphate dehydrogenase; KO, knockout; Veh, vehicle.

The 98 Nrf2-dependent meta cluster 2 genes were analyzed using IPA for functional enrichment and annotation. The most enriched pathways by these genes are the superpathway of cholesterol biosynthesis (*P* = 1.98 × 10^−12^) and cholesterol biosynthesis I (*P* = 5.52 × 10^−12^) (Table 3). Due to genes being regulated by D3T in meta cluster 1 as well as meta cluster 2, these two clusters were combined and analyzed for gene ontology using DAVID. DAVID analysis indicated that the KEGG pathways of terpenoid backbone synthesis (Fig. 3A) and steroid biosynthesis (Fig. 3B) were both highly enriched by D3T-responsive genes. Gene expression changes determined by microarray were validated by qPCR measurement of RNA transcripts (Fig. 3C). Of the 18 genes in the two cholesterol biosynthetic KEGG pathways, 10 genes were confirmed by qPCR to be uniquely regulated in the presence of D3T. Those genes are highlighted in yellow and overlaid onto the KEGG pathways in Fig. 3, A and B. Expression changes of eight genes in the cholesterol biosynthetic KEGG pathways regulated by D3T in Wt mouse liver were reversed in Nrf2-null tissue, indicating a requirement for Nrf2 in their response to D3T. To evaluate the efficacy of CDDO-Im in regulating cholesterol biosynthetic genes we determined to be unique to D3T-treatment, we examined the CDDO-Im dose-response relationship for *Lss* and lanosterol 14 *α*-demethylase (*Cyp51*). CDDO-Im, regardless of dose tested, had no effect on the expression of either of these genes (Fig. 3D), supporting their classification in meta cluster 2. Immunoblot analysis further confirmed both the Nrf2 dependence and the D3T-unique upregulation of LSS and CYP51 protein levels (Fig. 3E).

**TABLE 3:** Nrf2-dependent IPA canonical pathways enriched by genes responding uniquely to D3T treatment

D3T-Regulated Canonical Pathway	*P* Value	Genes
Superpathway of cholesterol biosynthesis	1.98 × 10^−12^	8 of 27
Cholesterol biosynthesis I	5.52 × 10^−12^	6 of 13

Meta cluster 3 (Table 2) contains 745 genes that responded uniquely to CDDO-Im. Remarkably, changes in the expression of 649 genes (87%) were dependent on Nrf2, indicating that CDDO-Im is a more selective activator of Keap1-Nrf2 signaling than D3T. Similar to meta cluster 2, we evaluated the response of the 297 Nrf2-dependent upregulated genes in meta cluster 3 to D3T. Of these, only 37 (13%) showed a level of response greater than 1.25-fold. In contrast, the expression of 243 genes (82%) was unchanged in the presence of D3T, indicating that the genes in this cluster are uniquely regulated by CDDO-Im and not a statistical artifact.

**Fig. 4.**
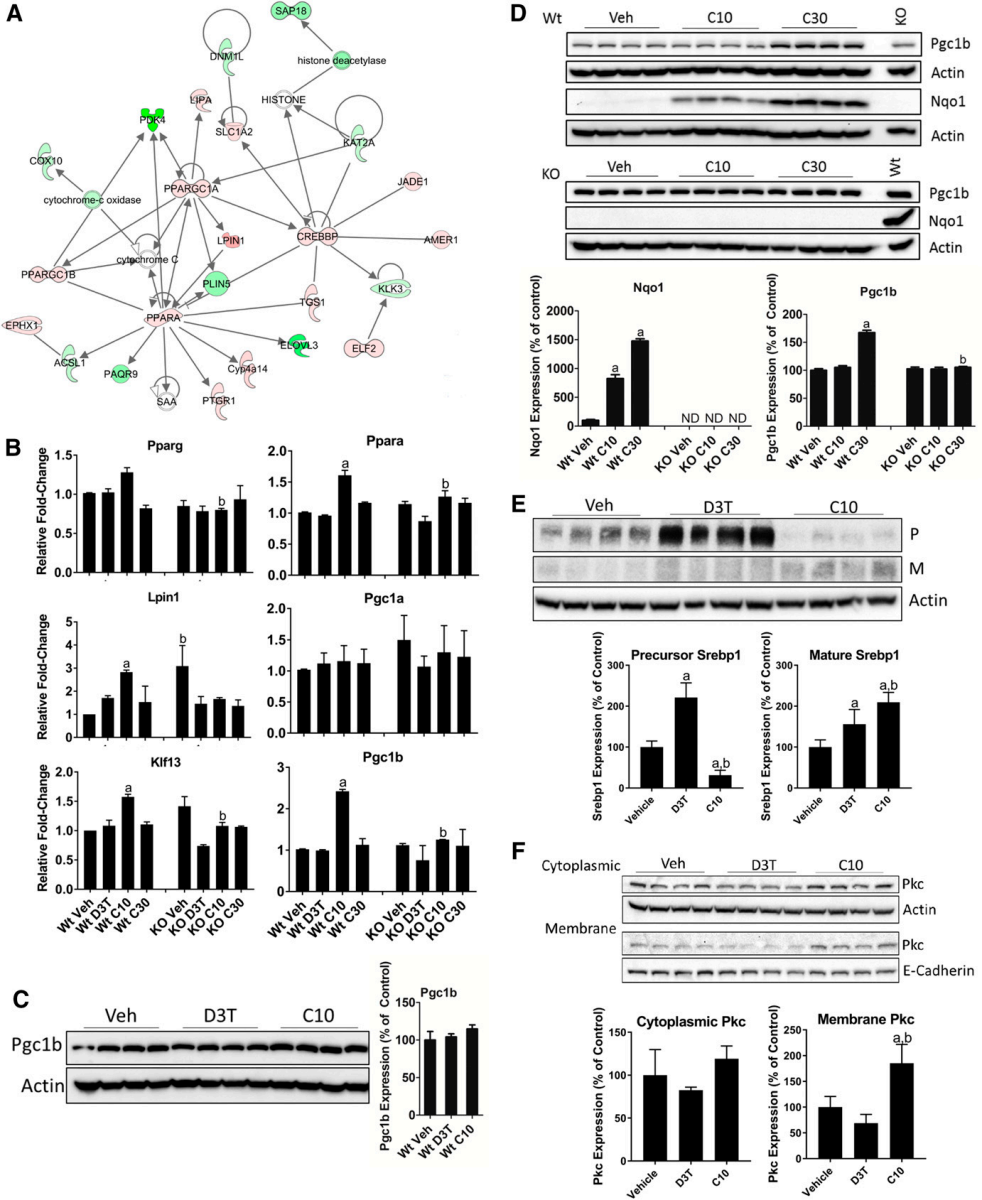
CDDO-Im selectively regulates lipid metabolism. (A) IPA of Nrf2-dependent genes unique to CDDO-Im (meta cluster 3, Table 2) combined with the common response genes (meta cluster 1, Table 2) identified the lipid metabolism pathway. Red nodes are upregulated, green nodes are downregulated, and the color intensity of each node indicates the magnitude of regulation as determined by microarray. (B) Measurement by qPCR of levels of RNA of the indicated genes. Animals were treated as described in the *Materials and Methods* with doses of 300 *μ*mol/kg b.wt. D3T or 3, 10, or 30 *μ*mol/kg b.wt. CDDO-Im. Values are expressed as the average fold-change ± S.E.M. in the liver of Wt or Nrf2-null (KO) mice (*n* = 2 independent experiments of four animals/treatment group per genotype each). Expression of *Gapdh*, which was unchanged by treatment or genotype, was used for normalization. Results were analyzed using a two-way ANOVA followed by Tukey’s multiple-comparisons test. ^a^*P* < 0.05 relative to the Wt vehicle; ^b^*P* < 0.05 relative to the Wt sample of the matching treatment and dose. (C) PGC1B expression in 50 *μ*g total liver protein from Wt mice treated with 300 *μ*mol/kg b.wt. D3T or 10 *μ*mol/kg b.wt. CDDO-Im. *β*-actin, which was unchanged by treatment or genotype, was used as a loading control. Values are expressed as the average percentage of the Wt vehicle control, which was set to 100% ± S.D. in the liver of Wt mice (*n* = 4/treatment group). Results were analyzed using a one-way ANOVA. (D) PGC1B and NQO1 expression in 50 *μ*g total liver protein from Wt or Nrf2-null mice treated with 10 or 30 *μ*mol/kg b.wt. CDDO-Im. *β*-actin, which was unchanged by treatment or genotype, was used as a loading control; data were normalized to a common sample loaded on both gels to allow for comparisons between genotypes. A sample from the 30 *μ*mol/kg b.wt. CDDO-Im–treated Wt tissue, labeled “Wt,” was loaded on the KO gels to serve as an NQO1 antibody detection control. Values are expressed as the average percentage of the Wt vehicle control, which was set to 100% ± S.D. in the liver of Wt or Nrf2-null (KO) mice (*n* = 4/treatment group per genotype). Results were analyzed using a one-way ANOVA followed by Dunnett’s multiple-comparisons test (NQO1) or by two-way ANOVA followed by Tukey’s multiple-comparisons test (PGC1B). ^a^*P* < 0.05 relative to the Wt vehicle (Veh); ^b^*P* < 0.05 relative to the Wt sample of the matching treatment and dose. (E) SREBP1 expression) in 50 *μ*g total liver protein from Wt mice treated with 300 *μ*mol/kg b.wt. D3T or 10 *μ*mol/kg b.wt. CDDO-Im. *β*-actin, which was unchanged by treatment, was used as a loading control. Values are expressed as the average percentage of the Wt vehicle control, which was set to 100% ± S.D. in the liver of Wt mice (*n* = 4). Results were analyzed using a one-way ANOVA followed by Tukey’s multiple-comparisons test. ^a^*P* < 0.05 relative to the vehicle; ^b^*P* < 0.05 relative to the D3T-treated sample. (F) Localization of PKC in 50 *μ*g cytoplasmic or membrane liver protein fractions from Wt mice treated with 300 *μ*mol/kg b.wt. D3T or 10 *μ*mol/kg b.wt. CDDO-Im. *β*-actin and E-cadherin, which were unchanged by treatment, were used as loading controls and specific markers of cytoplasmic and membrane fractions, respectively. Values are expressed as the average percentage of the vehicle control, which was set to 100% ± S.D. (*n* = 4). Results were analyzed using a one-way ANOVA followed by Tukey’s multiple-comparisons test. ^a^*P* < 0.05 relative to the vehicle; ^b^*P* < 0.05 relative to the D3T-treated sample. ANOVA, analysis of variance; *Gapdh*, glyceraldehyde 3-phosphate dehydrogenase; KO, knockout; M, mature form; ND, no protein was detected above background; P, precursor; Veh, vehicle.

The 649 Nrf2-dependent genes from meta cluster 3, along with the Nrf2-dependent genes from meta cluster 1, were analyzed for functional pathway enrichment using IPA (Fig. 4A). The pathway most enriched by these genes was lipid metabolism. To independently confirm this observation, we measured RNA transcripts of selected genes encoding top-level transcription factors and coactivators of lipid metabolism and *β*-oxidation (Fig. 4B). By qPCR, peroxisome proliferator *α* (*Ppara*), lipin 1 (*Lpin1*), Kruppel-like factor 13, and peroxisome proliferator *γ* coactivator 1*β* (*Pgc1b*) were all uniquely regulated by CDDO-Im. Based on the results of Figs. 1 and 2, we were surprised to observe that 30 *μ*mol/kg b.wt. CDDO-Im treatment did not increase the RNA expression of the lipid metabolic regulators to a level greater than the response to 10 *μ*mol/kg b.wt. CDDO-Im (Fig. 4B). This altered RNA dose response was also observed for many other genes, including the top 20 upregulated genes of meta cluster 3 (Table 4). Of the 649 Nrf2-dependent genes in meta cluster 3, the expression of 514 genes (79%) achieved maximal induction or repression in response to 10 *μ*mol/kg b.wt. CDDO-Im. Only 26 (4%) and 109 (17%) of these Nrf2-dependent genes reached maximal response by 3 or 30 *μ*mol/kg b.wt. CDDO-Im, respectively.

**TABLE 4:** CDDO-Im RNA expression dose response for the highest expressing Nrf2-dependent meta cluster 3 genes

Probe Set Identifier	Gene Symbol	Fold-Change		
		C3	C10	C30
1417168_a_at	*Usp2*	−1.43	12.84	1.00
1456225_x_at	*Trib3*	1.25	6.49	1.41
1416432_at	*Pfkfb3*	−1.45	4.78	1.45
1451548_at	*Upp2*	−1.14	3.91	−2.06
1452026_a_at	*Pla2g12a*	1.30	3.83	−1.01
1448667_x_at	*Tob2*	1.67	3.56	1.77
1450703_at	*Slc7a2*	1.09	3.53	1.45
1416773_at	*Wee1*	−1.23	3.28	1.15
1445843_at	*Chd2*	2.24	3.20	2.07
1449409_at	*Sult1c2*	1.31	3.17	−1.78
1460521_a_at	*Obfc2a*	1.20	3.07	1.91
1428730_at	*Krit1*	2.54	2.92	1.92
1454617_at	*Arrdc3*	1.08	2.89	−1.40
1451716_at	*Mafb*	1.43	2.86	1.18
1421852_at	*Kcnk5*	1.63	2.78	1.11
1436830_at	*Marveld1*	1.03	2.78	−1.15
1450743_s_at	*Syncrip*	1.80	2.74	2.31
1440146_at	*Vps13a*	1.19	2.72	1.39
1429432_at	*Bat2d*	1.76	2.63	2.01
1431098_at	*Clip1*	2.01	2.63	2.16

Because *Pgc1b* was clearly unique to CDDO-Im and had previously been reported to be transcriptionally regulated by Nrf2 (Chorley et al., 2012), we examined PGC1B protein expression in the liver of Wt mice treated with equally efficacious doses of D3T and CDDO-Im. Surprisingly, PGC1B protein levels were not altered by either D3T or CDDO-Im (Fig. 4C). To further explore dose-response relationships of this cluster, we evaluated the expression of PGC1B protein in mouse liver treated with 10 or 30 *μ*mol/kg b.wt. CDDO-Im (Fig. 4D). Despite our observations that *Pgc1b* RNA expression reached a maximal response to 10 *μ*mol/kg b.wt. CDDO-Im, 30 *μ*mol/kg b.wt. CDDO-Im treatment led to the enhanced induction of PGC1B protein expression above that which would have been expected based on the RNA response to a lower CDDO-Im dose. Although this observation is not fully understood, previous reports suggest that oxidizing intracellular conditions result in markedly impaired mRNA translation, through the inhibition of elongation factor 2, which can be rescued through supplementation with antioxidants (Chio et al., 2016). The ability of CDDO-Im to potently and selectively regulate Nrf2-dependent antioxidant functional pathways may result in enhanced mRNA translation. Due to enhanced protein expression, these data support the continued use of 30 *μ*mol/kg b.wt. CDDO-Im for future cancer chemoprevention and lipid metabolic studies.

The induction of lipid metabolic pathways in response to CDDO-Im is consistent with the isoform-specific activity of Srebp1c, which is known to transcriptionally regulate genes involved in these pathways (Horton et al., 2002). We measured SREBP1C expression in the liver of D3T– and 10 *μ*mol/kg b.wt. CDDO-Im–treated Wt mice (Fig. 4E). In agreement with the CDDO-Im–mediated induction of lipid metabolic regulators, we observed significantly elevated levels of the mature form of SREBP1C in response to CDDO-Im relative to the levels observed in response to D3T.

In addition to SREBP1C, upstream activation of PKC has also been demonstrated to mediate both SREBP1C and PPARA activity to further drive metabolic flux toward lipogenic programs (Gray et al., 2005; Yamamoto et al., 2010). Because PKC localization to the plasma membrane is a reliable marker for its activity (Mochly-Rosen, 1995), we measured the enrichment of PKC in the cytoplasmic and membrane fractions in the liver of Wt mice treated with 300 *μ*mol/kg b.wt. D3T or 10 *μ*mol/kg b.wt. CDDO-Im (Fig. 4F). We observed significantly elevated PKC levels in the membrane fraction only in response to CDDO-Im, suggesting that CDDO-Im–mediated activation of PKC, in combination with its effects on NRF2 and PPARA, may contribute to the treatment-specific regulation of lipid metabolic pathways.

## Discussion

We systematically compared the pharmacodynamics of two Nrf2 chemical activators, D3T and CDDO-Im, and defined the gene regulatory responses elicited by each compound. D3T and CDDO-Im are prototypes of the dithiolethione and synthetic triterpenoid classes of Nrf2-activating agents, respectively. Previous reports have suggested that different Nrf2-activating mechanisms may lead to distinct gene responses (Tran et al., 2009; Yates et al., 2009; To et al., 2015). Due to the presence of unique pharmacophores, we hypothesized that D3T and CDDO-Im may lead to the regulation of treatment-specific gene sets and functional pathways, potentially facilitating or confounding the therapeutic efficacies of these compounds. Inherent differences in potency across Nrf2-activating compounds have made direct comparisons between chemical classes problematic. Moreover, differences in drug disposition can potentially lead to differences in the duration of Nrf2 activity, resulting in unique Nrf2-dependent signaling events. To efficiently assess the fundamental pharmacodynamics of D3T and CDDO-Im, we minimized these variables by establishing equally efficacious doses of each compound and by administering the treatments intermittently over several doses to provide a consistent level of Nrf2-activity. Based on the response of well characterized Nrf2 targets, this experimental design appeared to lessen differences in potency and the resulting duration of Nrf2 activation. As expected, genes involved in the Nrf2 antioxidant response were commonly regulated by equally efficacious doses of D3T and CDDO-Im to a similar effect.

Remarkably, we observed unique Nrf2-dependent gene subsets regulated by each treatment that were associated with enrichment of different lipid metabolic processes. D3T enriched cholesterol metabolism, while CDDO-Im enriched lipid synthesis and *β*-oxidation pathways. Because both compounds target Keap1, the mechanism underlying this treatment-specific gene regulation is unclear.

Initially, we had hypothesized that distinct sites of Keap1 cysteine modification could result in treatment-specific gene regulation. In the yeast, Yap1 cysteine residues are differentially modified in the presence of different compounds, resulting in treatment-specific gene regulation (Ouyang et al., 2011). Indeed, early reports on the regulation of Keap1/Nrf2 by site-directed mutagenesis studies using zebrafish reported preferential modification of Keap1 Cys151 by D3T, while CDDO-Im targeted Cys273 and Cys288 (Takaya et al., 2012). Recent reports, however, indicate that Keap1 Cys151 is the preferred target for both D3T and CDDO-Im (Saito et al., 2015). It is noteworthy that this study used site-directed mutagenesis to establish the requirement of Keap1 cysteine residues for the stabilization of total Nrf2 protein, not nuclear Nrf2 protein accumulation or gene transcription. Thus, although it seems likely that Nrf2-activating agents elicit unique gene responses independent of Keap1, further study of this topic appears warranted.

An alternative explanation and emerging concept in the regulation of metabolic networks is the role of coactivators and corepressors in coordinating the activities of multiple nuclear receptors and their functions (Lin et al., 2005; Kriebs et al., 2017). In relation to the D3T- and CDDO-selective enrichments observed here, the potential for differential regulation of SREBP isoforms may be important. SREBP1C, the predominant isoform in adult mouse liver, preferentially regulates genes required for fatty acid synthesis, including the rate-limiting enzyme in triglyceride and phospholipid synthesis glycerol-3-phosphate acyltransferase. SREBP2, however, is a preferential regulator of cholesterol biosynthesis genes (Horton et al., 2002). Selective regulation of these SREBPs in response to D3T and CDDO-Im represents a plausible mechanism whereby Nrf2-activating agents may exert unique effects on gene transcription and pathway regulation. To test this hypothesis, we attempted to measure SREBP2 RNA and protein expression in D3T-treated mouse liver. Despite attempts with two different SREBP2 antibodies, we failed to detect SREBP2 protein and did not observe any change in *Srebp2* RNA expression in response to either treatment (data not shown).

In contrast to our findings, previous reports evaluating the effect of D3T in the liver of rodents have observed repressed *Srebp* expression, indicating a likely downregulation of downstream SREBP transcriptional targets (Kwak et al., 2003; Huang et al., 2006). We believe that differences in the experimental designs of these studies could account for such effects. Kwak et al. (2003) treated mice with D3T for 24 hours with a dose 1.5 times greater than what was used here, which likely represents an acute, maximally tolerated dose of D3T rather than the intermittent therapeutic dose used here. Huang et al. (2006) treated rats with D3T, suggesting species differences. For example, D3T treatment in rats repressed *Insig1*, a known mediator of SREBP stability, indicating that the effect of D3T on *Srebp* gene expression may not be reflected in SREBP protein levels or activity (Huang et al., 2006). Moreover, chronic treatment of both rats and dogs with oltipraz, a substituted analog of D3T, resulted in significantly elevated total cholesterol (Crowell et al., 1997). Although the effects of D3T and Nrf2 on cholesterol metabolism are complex, we believe that this previously reported observation of elevated cholesterol in vivo lends support to our observations that D3T may act to upregulate the cholesterol biosynthetic pathway.

In contrast to D3T, we observed upregulation of several top-level transcription factors and coactivators of lipid metabolism in response to CDDO-Im, indicating that CDDO-Im may affect lipid metabolism through the unique regulation of alternative transcriptional and signaling pathways beyond Keap1/Nrf2. As a key regulator of lipid metabolic genes, regulation of SREBP1C in response to CDDO-Im could account for these observations. We observed a significant upregulation of the mature protein form of SREBP1C in response to both treatments, albeit the expression of mature SREBP1C was significantly higher in the liver of CDDO-Im–treated mice relative to those treated with D3T. Furthermore, we also observed the activation of PKC only in response to treatment with CDDO-Im. The unique regulation of PKC is particularly interesting because PKC directly phosphorylates Nrf2 (Huang et al., 2002; Bloom and Jaiswal, 2003) and PKC signaling appears to enhance the activity of SREBP1C and PPARA, both of which contribute to the lipid metabolic programs regulated by CDDO-Im (Gray et al., 2005; Yamamoto et al., 2010). In addition to these effects, CDDO-Im is also a direct PPARG agonist (Place et al., 2003) and a regulator of PPARG coactivators such as *Pgc1b*. Thus, in considering the effects of CDDO-Im, one must understand the Nrf2-dependent gene regulatory effects as well as other drug-related activities. Although the regulation of these additional signaling pathways does not mechanistically link them to the unique regulation of lipid metabolism by CDDO-Im, it does allude to the potential importance of non-Nrf2 signaling modifiers and their effects on Nrf2-mediated gene regulatory outcomes. The identity and contributions of such signaling pathways on Nrf2-dependent gene transcription is an area that warrants more investigation.

Several studies have shown that Nrf2 negatively regulates fatty acid and triglyceride metabolism, with the absence of Nrf2 being associated with increased liver lipid accumulation (Chowdhry et al., 2013; Okada et al., 2013). These results are consistent with the beneficial effects of Nrf2 activation in the prevention of type 2 diabetes and metabolic disease (Shin et al., 2007; Tanaka et al., 2008; Chartoumpekis et al., 2011; Slocum et al., 2016). It is plausible that activation of Nrf2 provides protection against these diseases through the antagonism of fatty acid synthesis via transcriptional repression of both fatty acid synthase and acetyl-CoA carboxylase 1 (Shin et al., 2007). In addition, enhanced *β*-oxidation may account for reduced lipid accumulation in response to Nrf2 activation. We observed repressed acetyl-CoA carboxylase 2 (data not shown) and elevated *Ppara* expression in response to CDDO-Im treatment, both reliable biomarkers of elevated *β*-oxidation (Desvergne and Wahli, 1999; Chartoumpekis and Kensler, 2013). We also observed unique regulation of Pgc1b in the presence of CDDO-Im, which coactivates PPARA and contributes to enhanced *β*-oxidation and resistance to obesity (Kamei et al., 2003). Taken together, these data provide a weight of evidence supporting the beneficial effects of CDDO-Im to prevent high-fat diet–induced obesity (Shin et al., 2009; Slocum et al., 2016).

This study clearly demonstrates that two chemically distinct Nrf2 activators (D3T and CDDO-Im) regulate both common and unique genes, proteins, and pathways in the mouse liver. In addition to Nrf2-mediated chemical-specific targets, we also observed chemical-selective activation of additional signaling pathways. Future studies are necessary to understand the integration of signaling pathway cross-talk and coactivator/corepressor function into the key metabolic pathways that are regulated in a chemical-selective Nrf2-dependent manner. Without further knowledge of the effects identified in this study, the field of Nrf2 drug development will be limited to studies of individual compounds rather than mechanism-based chemical classes. The ongoing clinical use of Nrf2 activators for prophylactic and therapeutic applications underscores the importance of understanding the chemical-selective responses observed here.

## Abbreviations

aRNA: amplified RNA
CDDO-Im: 1-[2-cyano-3,12-dioxooleana-1,9(11)-dien-28-oyl]imidazole
D3T: 3*H*-1,2-dithiole-3-thione
DAVID: Database for Annotation, Visualization and Integrated Discovery
GST: glutathione *S*-transferase
IPA: Ingenuity Pathway Analysis
KEGG: Kyoto Encyclopedia of Genes and Genomes
LSS: lanosterol synthase
NQO: NAD(P)H:quinone oxidoreductase
Nrf2: NF-E2-related factor 2
PKC: protein kinase C
qPCR: quantitative polymerase chain reaction
SREBP: sterol regulatory element-binding protein
Wt: wild type.
